# Supplementary material for: Galacto-Oligosaccharides Increase the Abundance of Beneficial Probiotic Bacteria and Improve Gut Architecture and Goblet Cell Expression in Poorly Performing Piglets, but Not Performance
Source: Animals (Basel). 2023 Jan 8;13(2):230. doi: 10.3390/ani13020230 (PMC9854465; doi:10.3390/ani13020230)

Table S1. Composition of Faramate Milk Replacer Feed for Piglets (10 kg)

Crude protein (whey)	22 %	Vitamin B1	5 mg
Crude fibre	0 %	Vitamin B2	3 mg
Crude oils & fats	14 %	Vitamin B6	3 mg
Crude ash	7.5 %	Vitamin B12	60 µg
Lysine	2 %	Vitamin C	100 mg
Calcium	0.9 %	Nicotinic acid	20 mg
Sodium	0.5 %	Biotin	50 µg
Phosphorous	0.7 %	Pantothenic acid	10 mg
Butylated hydroxytoluene (Antioxidant)	150 mg	Copper (Copper sulphate pentahydrate)	10 mg
Citric acid (Preservative)	1000 mg	Iodine (Potassium iodide)	0.25 mg
Vitamin A (Alphatocopherol acetate)	25,000 iu	Iron (Ferrous sulphate monohydrate)	100 mg
Vitamin D3	10,000 iu	Manganese (Manganese sulphate monohydrate)	40 mg
Vitamin E	500 iu	Selenium (Sodium selenite)	0.4 mg
Vitamin K3	3 mg	Zinc (Zinc sulphate monohydrate)	50 mg

Table S2. Number of high-quality sequences obtained from each trial and section of the piglet GIT.

	Duodenum	Jejunum	Ileum	Caecum	Colon	Rectum	Total
**Trial 1**	93,842	78,741	82,920	111,020	70,940	83,411	**520,874**
**Trial 2**	144,112	169,032	172,104	122,170	83,598	101,245	**792,261**
**Trial 3**	62,097	75,177	98,644	131,896	188,297	90,018	**646,129**
**Trial 4**	66,485	68,400	86,519	74,596	65,549	59,596	**421,145**
**Total**	**366,536**	**391,350**	**440,187**	**439,682**	**408,384**	**334,270**	**2,380,409**

Table S3. Number of high-quality sequences clustered into OTUs obtained from each trial and section of the piglet GIT.

	Duodenum	Jejunum	Ileum	Caecum	Colon	Rectum	Total
**Trial 1**	377	277	540	635	544	789	**(1279)**
**Trial 2**	710	728	754	715	785	788	**(1726)**
**Trial 3**	537	354	338	552	751	698	**(1273)**
**Trial 4**	476	347	497	692	763	733	**(1216)**
**Total**	**(1062)**	**(1000)**	**(1182)**	**(1339)**	**(1474)**	**(1503)**	**(3281)**

Totals in parentheses are not arithmetic sums of columns or rows but the total number of OTUs obtained across samples for the GIT and for each trial since many OTUs are shared between samples.

Figure S1. Piglet weight at 24h *post-partum* correlates with end-of-study weight at day 21. (Linear modelling, $R^2 = 0.5$, $P < 0.001$ for non-GOS piglets, $R^2 = 0.48$, $P < 0.001$ for GOS fed piglets).

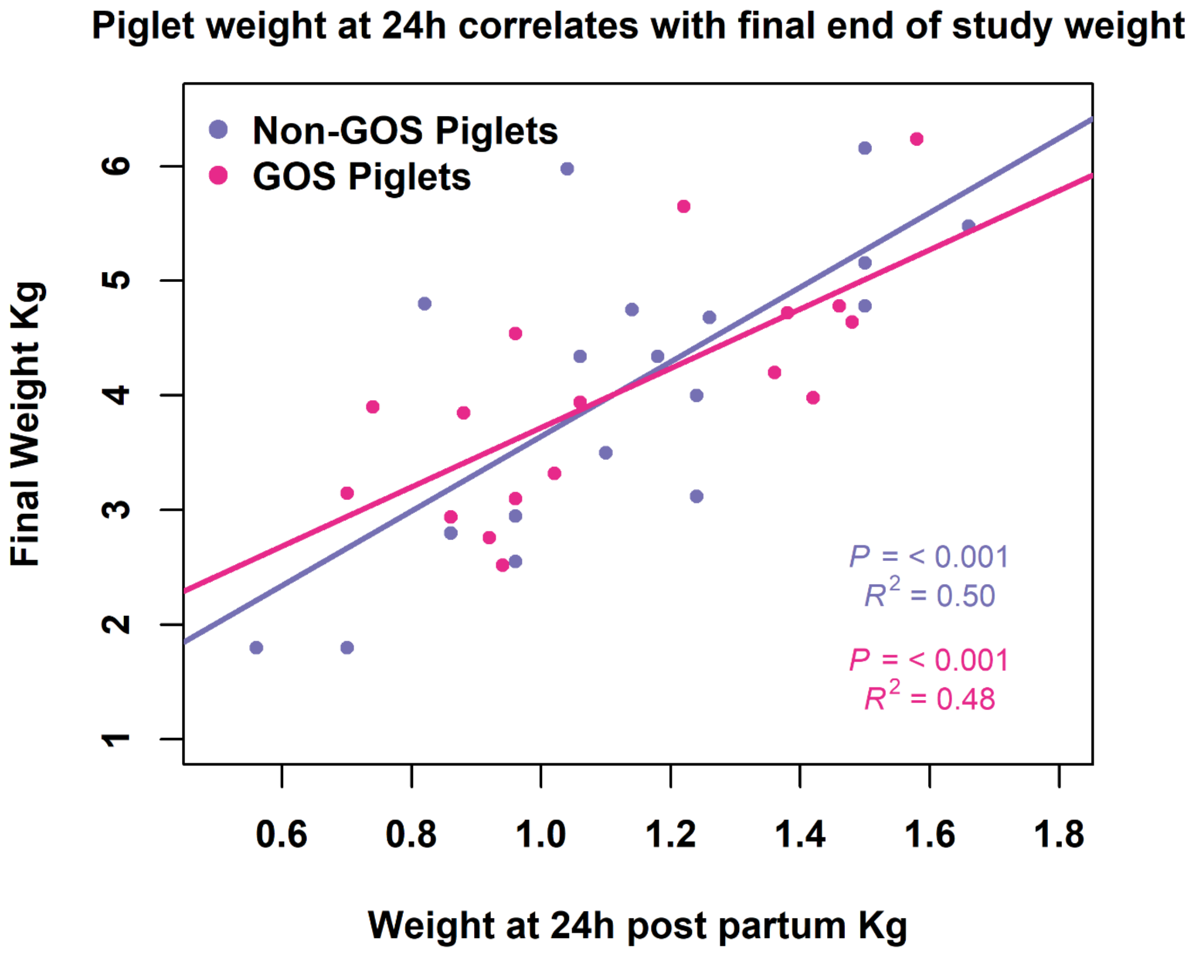

Figure S2. Daily feed intake for non-GOS fed piglets and GOS fed piglets for all trials days 1 to 21. Mean DFI not significantly different between treatments (*t*-test, $P = 0.802$).

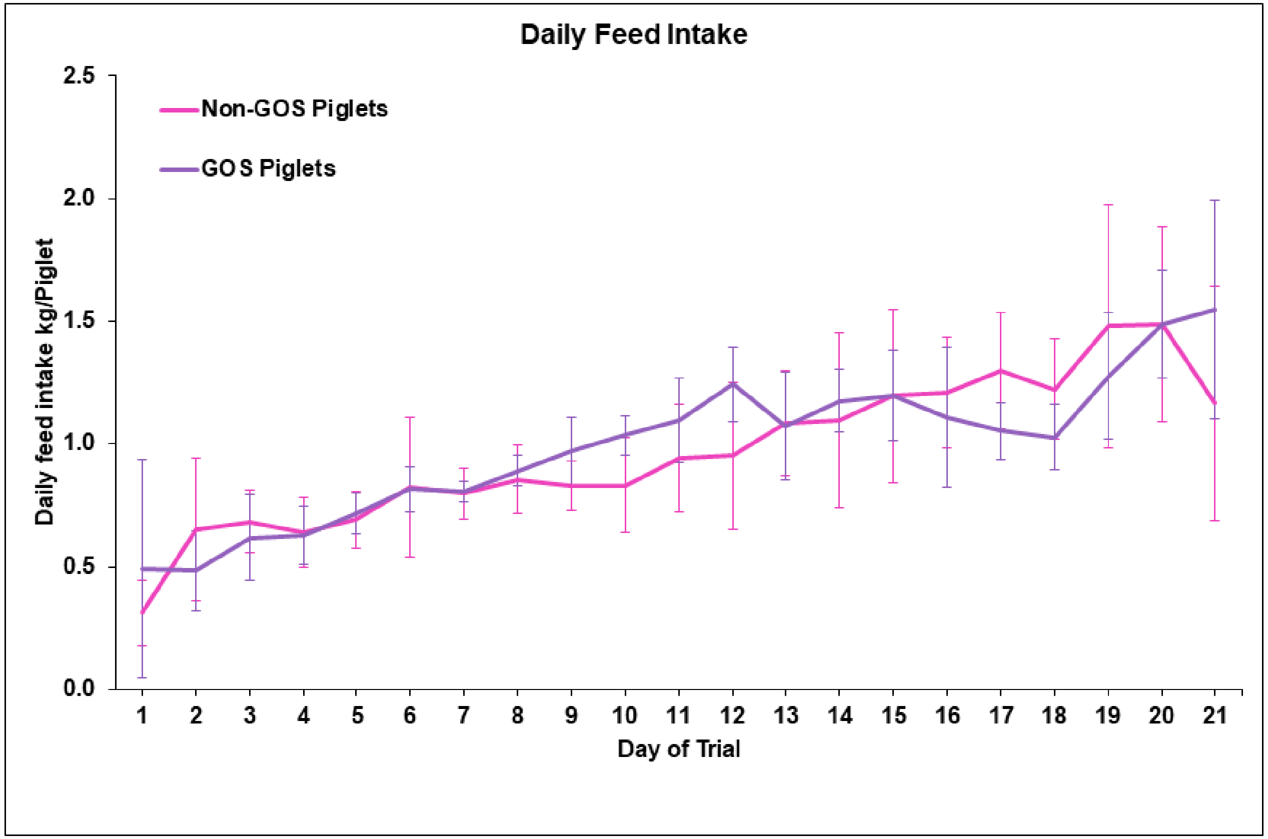

Figure S3A. Trial 1. β-diversity measures for non-GOS fed piglets and GOS fed piglets for each GIT sample.

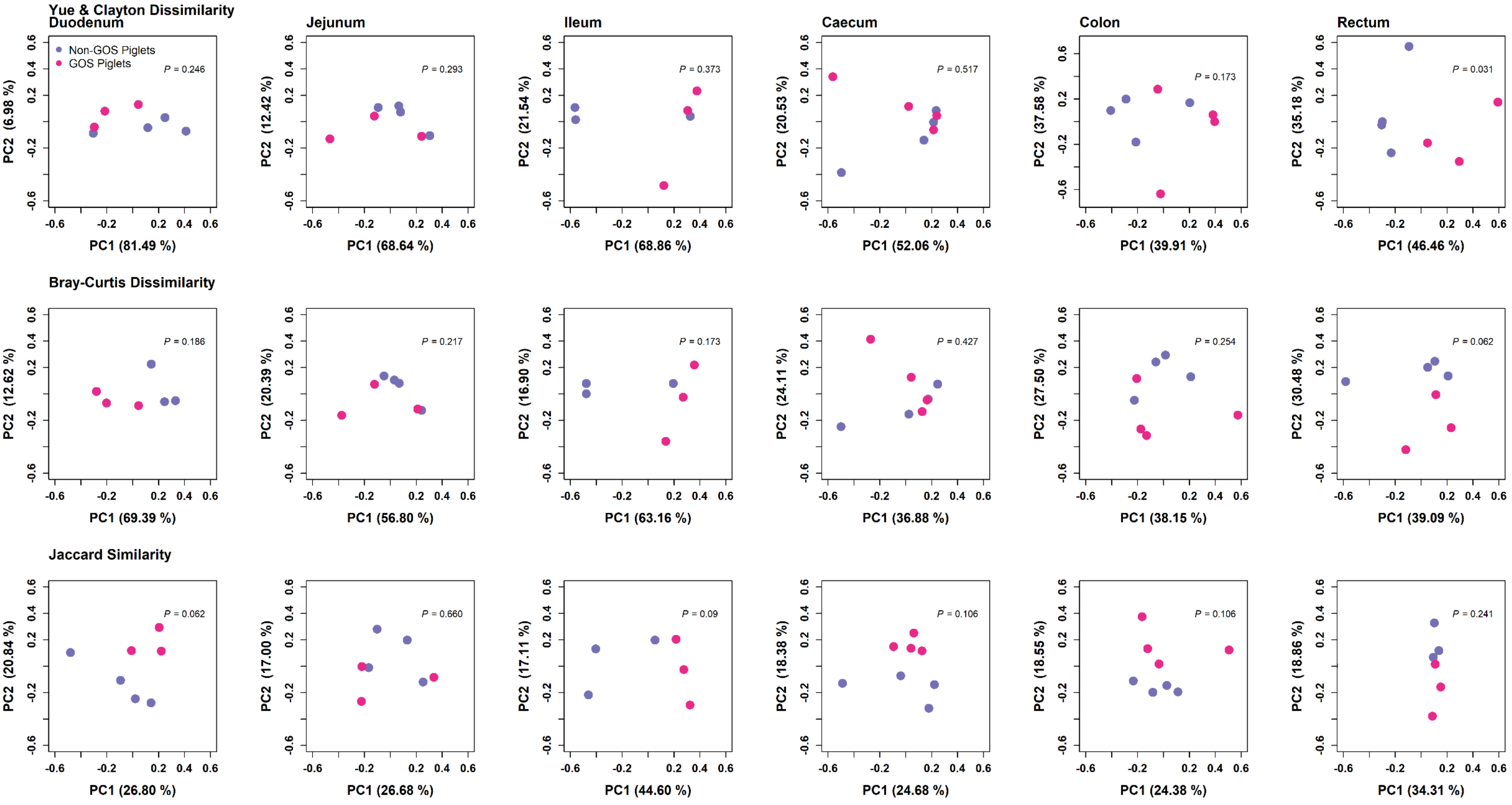

Figure S3B. Trial 2. β-diversity measures for non-GOS fed piglets and GOS fed piglets for each GIT sample.

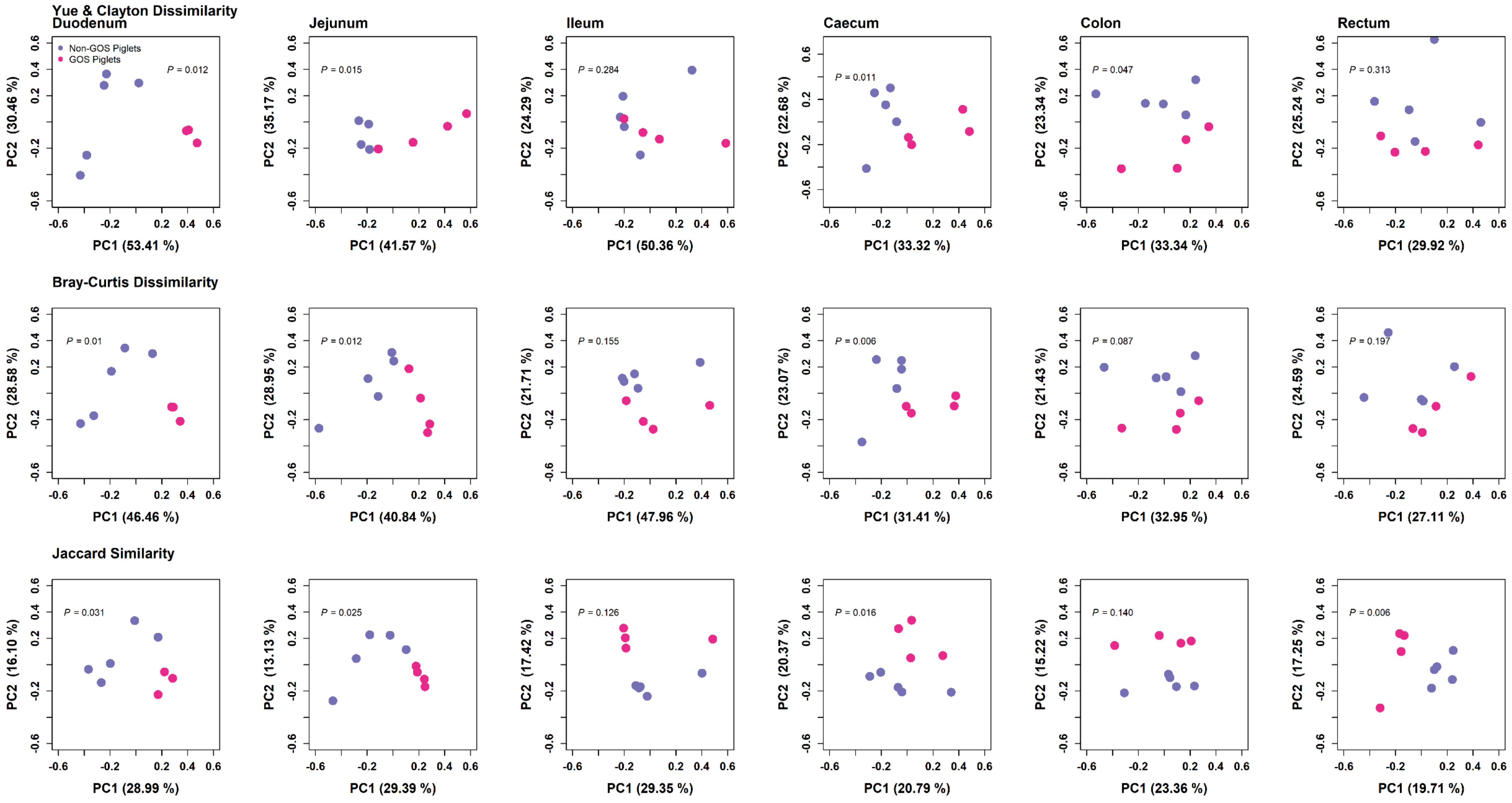

Figure S3C. Trial 3. β-diversity measures for non-GOS fed piglets and GOS fed piglets for each GIT sample.

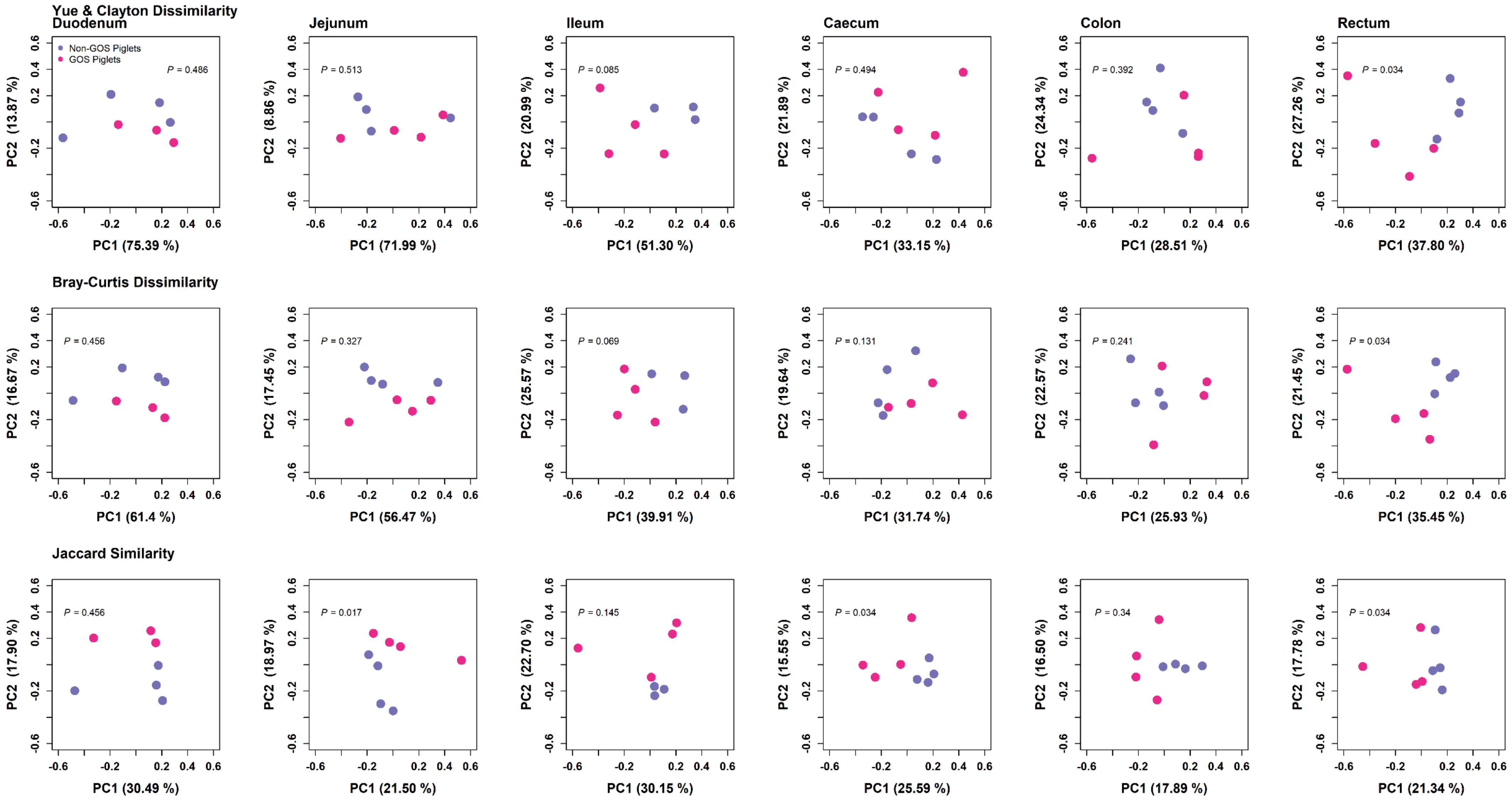

Figure S3D. Trial 4. β-diversity measures for non-GOS fed piglets and GOS fed piglets for each GIT sample.

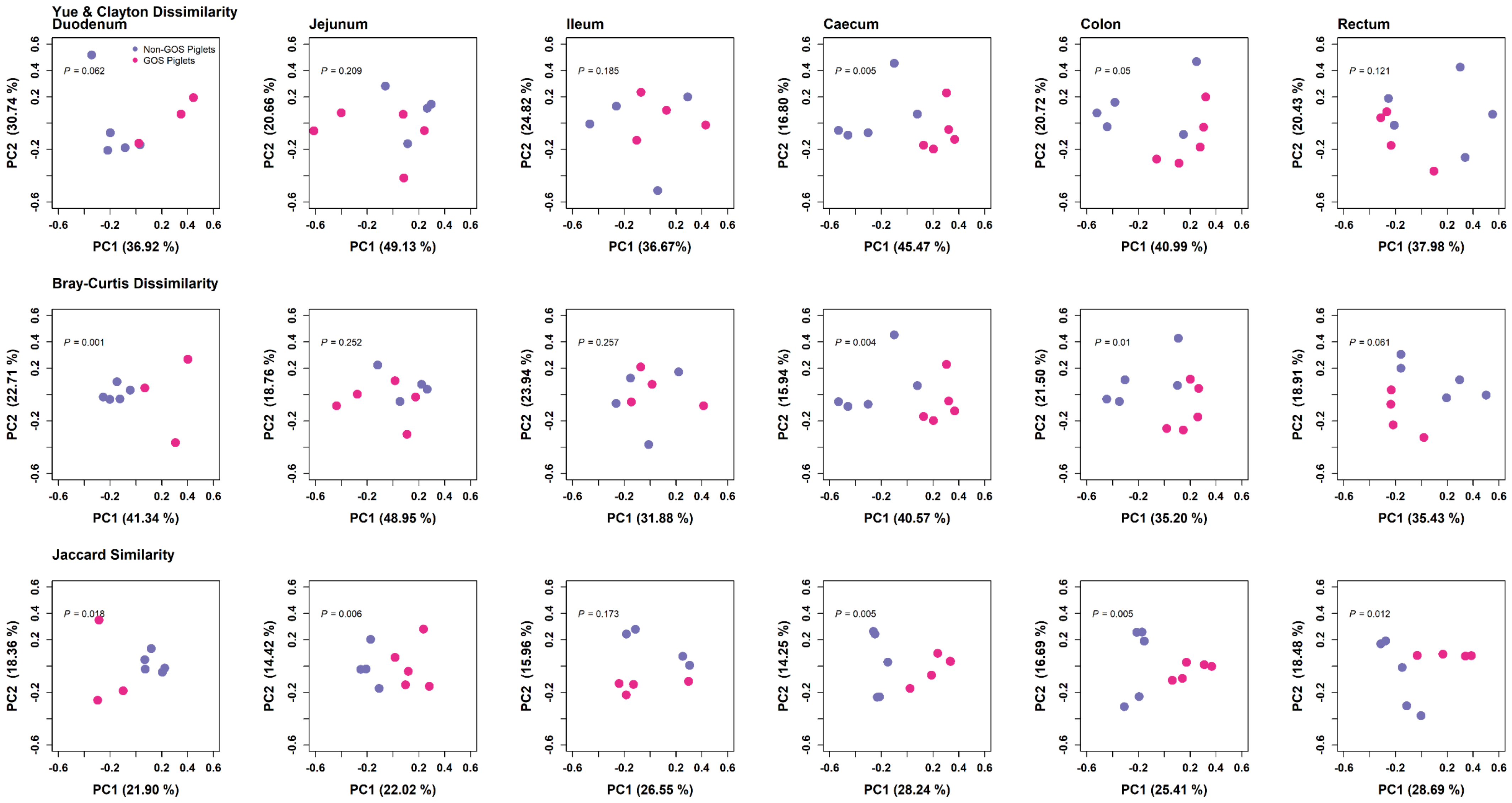

Figures S4A Trial 1. Significant differences in differential abundance of OTUs ascribed to taxa at genus level throughout the GIT for non-GOS and GOS fed piglets (* $P < 0.05$, ** $P < 0.01$, *** $P < 0.001$, LEfSe). UC = "Unclassified".

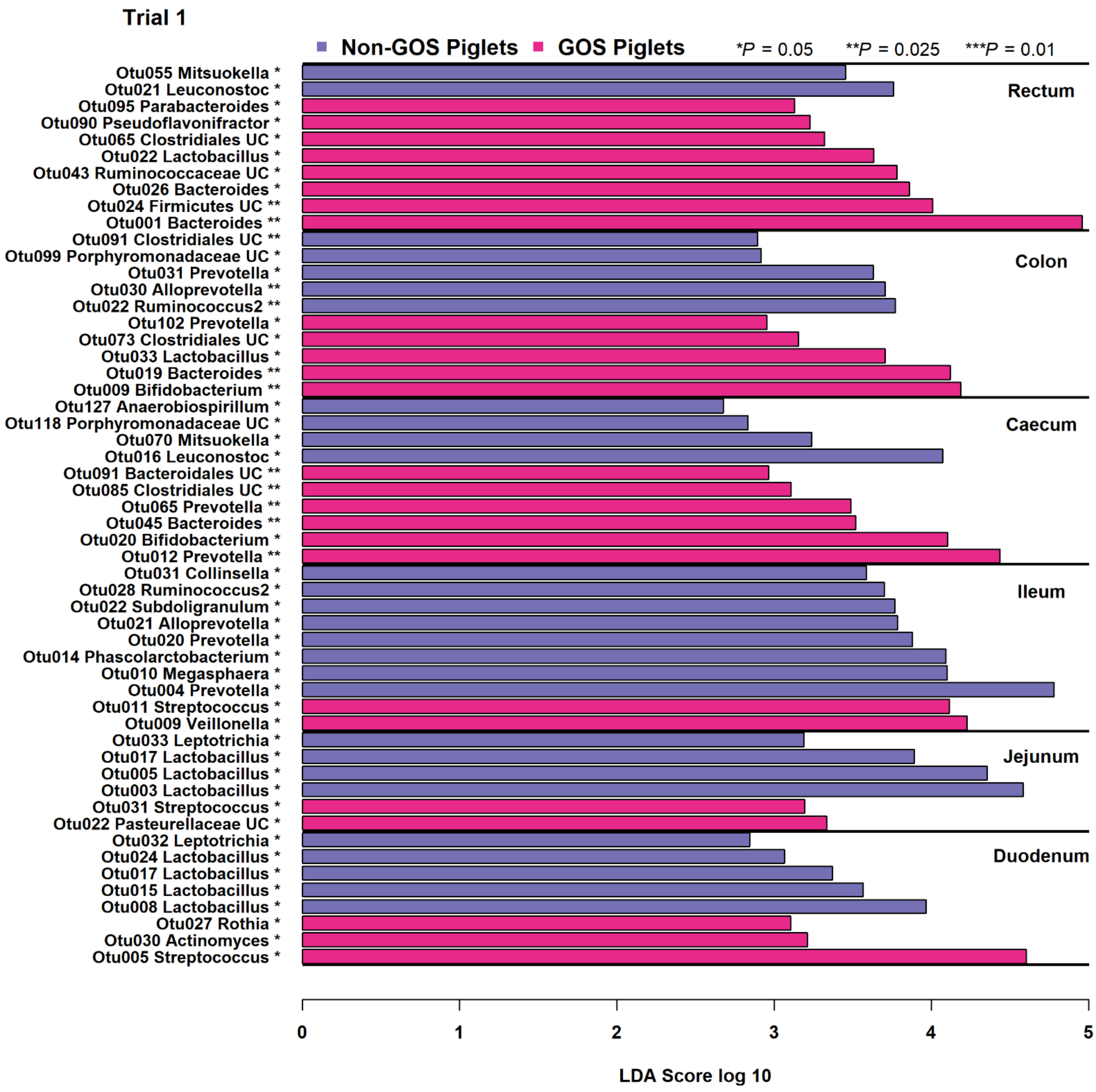

Figures S4B Trial 2. Significant differences in differential abundance of OTUs ascribed to taxa at genus level throughout the GIT for non-GOS and GOS fed piglets (* $P < 0.05$, ** $P < 0.01$, *** $P < 0.001$, LEfSe). UC = "Unclassified".

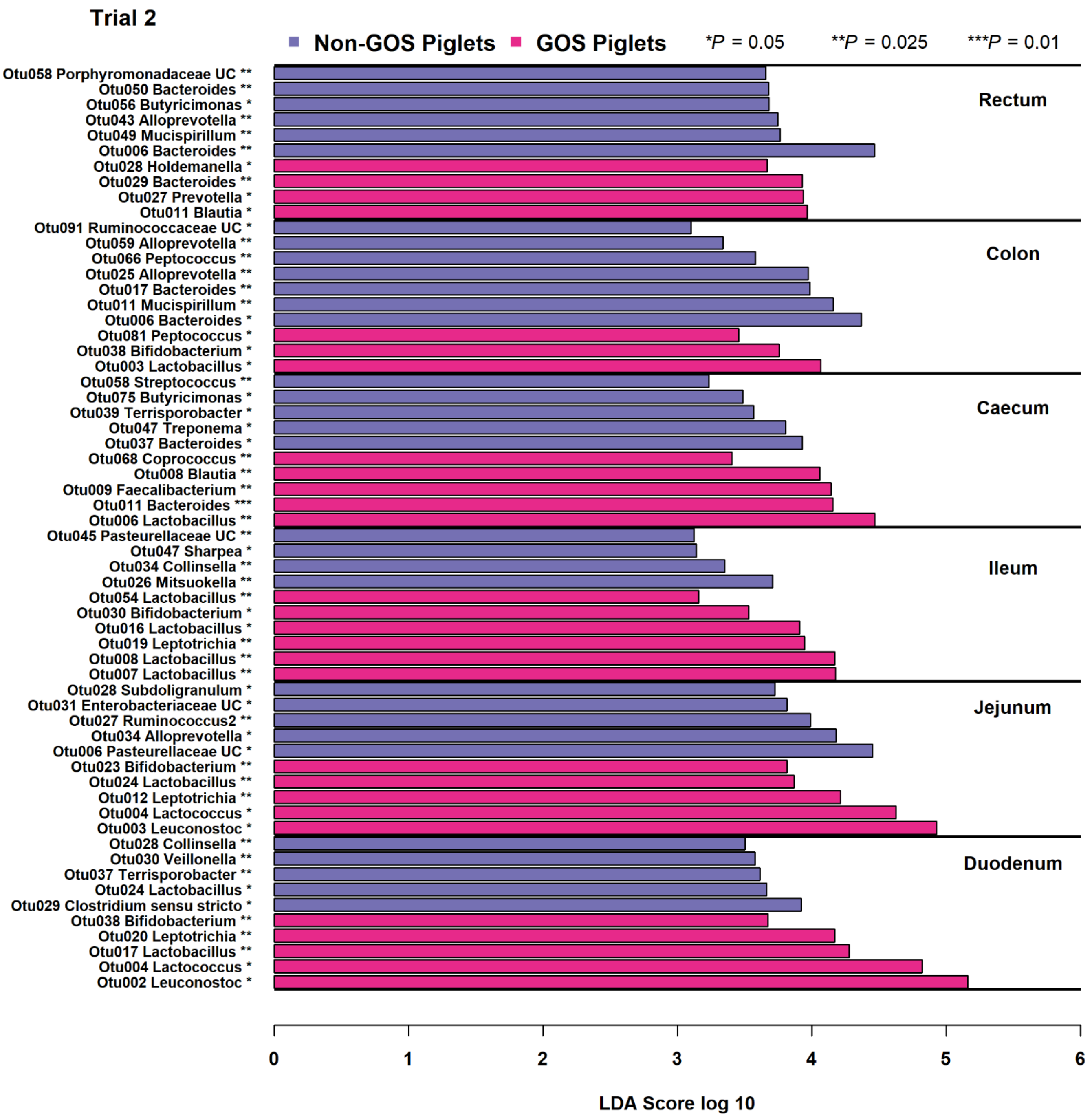

Figures S4C Trial 3. Significant differences in differential abundance of OTUs ascribed to taxa at genus level throughout the GIT for non-GOS and GOS fed piglets (* $P < 0.05$, ** $P < 0.01$, *** $P < 0.001$, LEfSe). UC = "Unclassified".

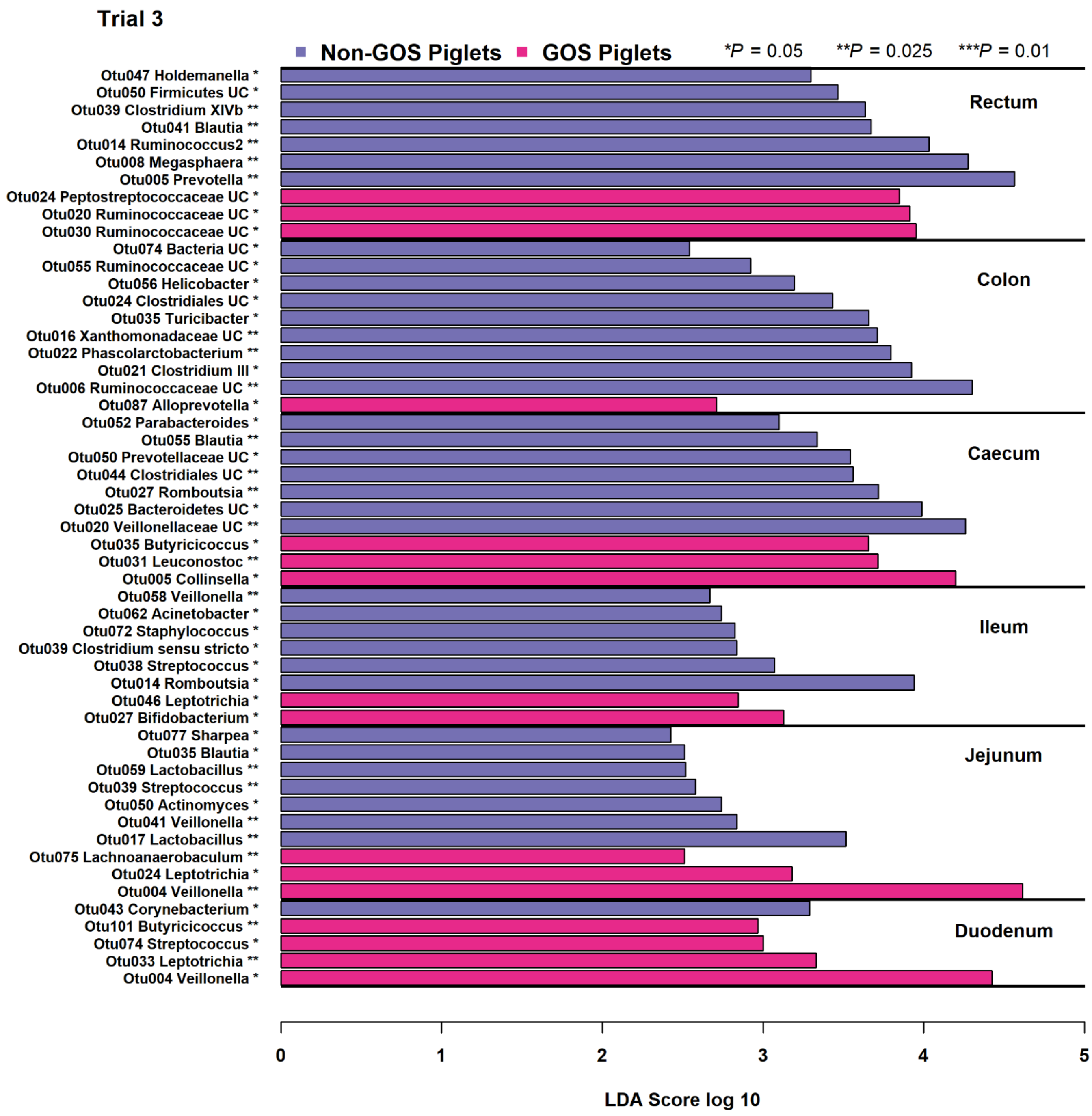

Figures S4D Trial 4. Significant differences in differential abundance of OTUs ascribed to taxa at genus level throughout the GIT for non-GOS and GOS fed piglets (* $P < 0.05$, ** $P < 0.01$, *** $P < 0.001$, LEfSe). UC = "Unclassified".

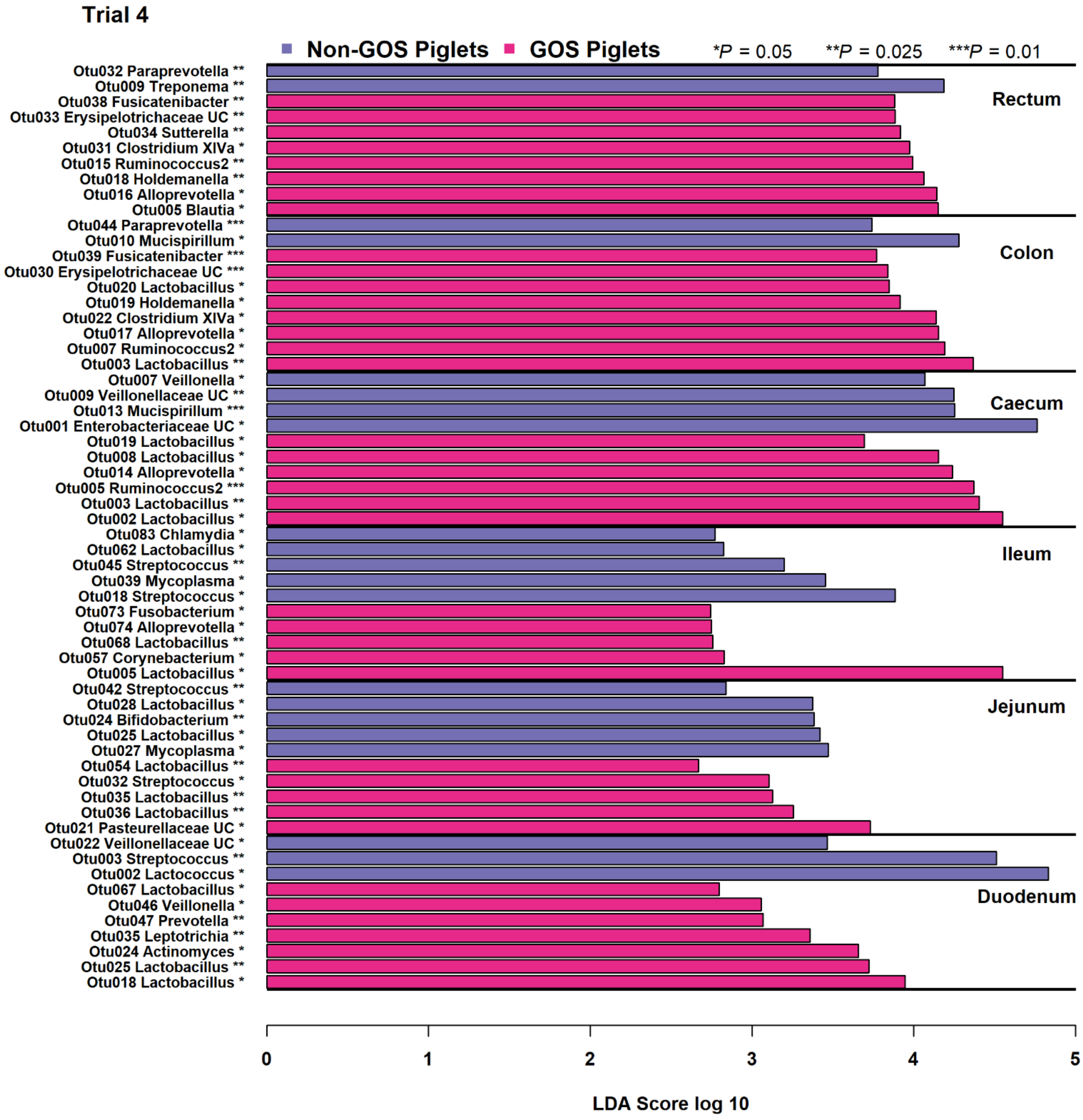

Figure S5. Correlation between jejunal and ileal villus area and height and jejunal, ileal, colonic and caecal crypt area and depth for all trials. Significant correlation between jejunal and ileal villus area and height and jejunal, ileal, colonic and caecal crypt area and depth ($P < 0.001$, in each case, linear modelling). Jejunal villus height significantly greater for GOS fed piglets as opposed to non-GOS fed piglets ($P = 1.7 \times 10^{-11}$) and ileal villus height ($P = 2.1 \times 10^{-8}$), caecal crypt depth ($P = 1.8 \times 10^{-4}$) and colonic crypt depth ($P = 0.008$, Wilcoxon rank sum exact tests). No significant differences in jejunal and ileal crypt depth between non-GOS fed and GOS fed piglets.

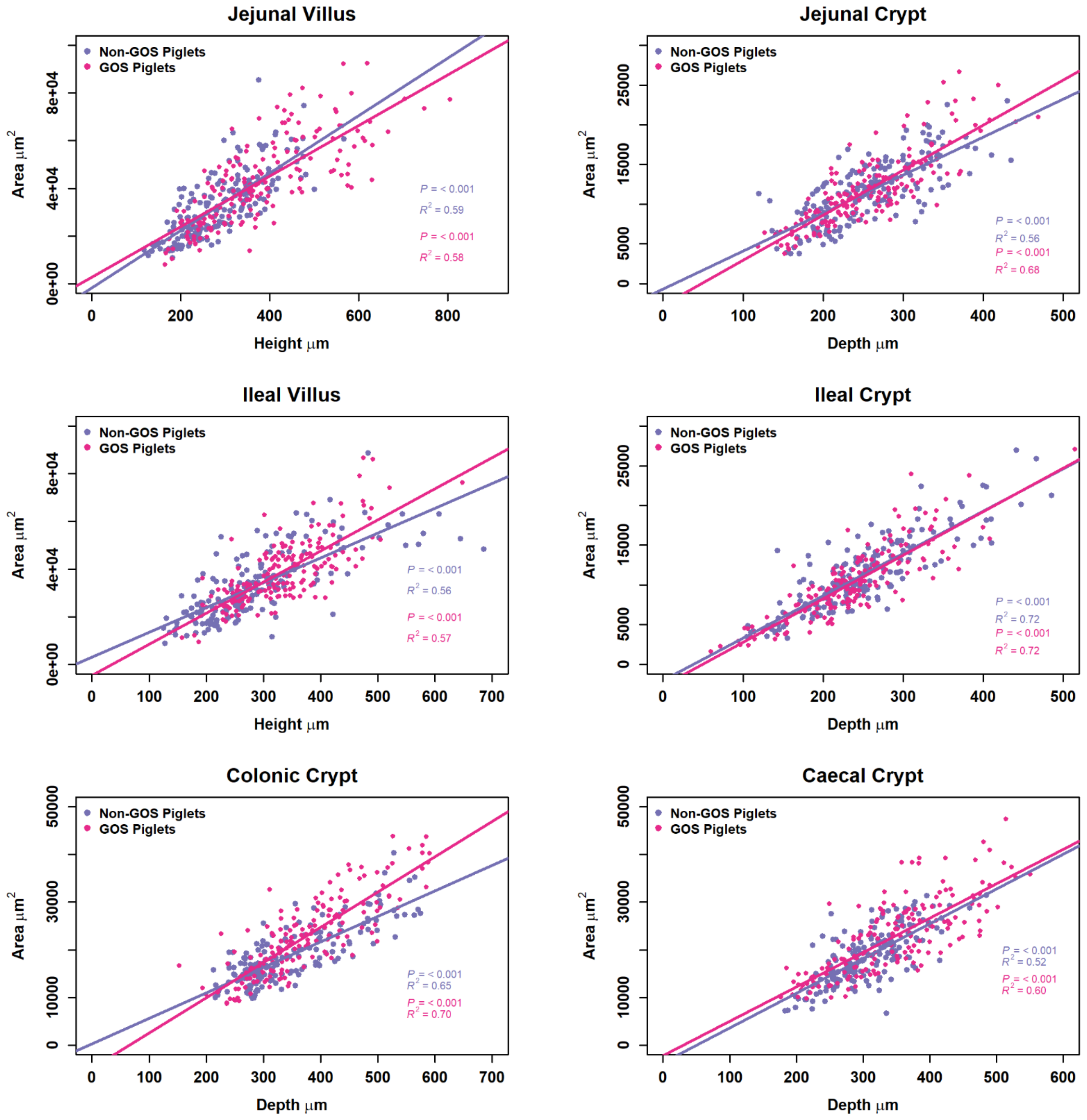

Figure S6. Correlation between jejunal and ileal villus area, jejunal, ileal, caecal and colonic crypt area with GC density expressed as number of GCs per mm^2 for all trials. Significant correlation between jejunal and ileal villus area, jejunal, ileal, caecal and colonic crypt area with GC density ($P < 0.001$ in each case, linear modelling). Number of GCs per mm^2 GIT tissue significantly higher in GOS fed piglets than non-GOS fed piglets for the jejunal villus ($P = 7.4 \times 10^{-6}$), the jejunal crypt ($P = 1.4 \times 10^{-4}$), the ileal villus ($P = 2.8 \times 10^{-5}$), the ileal crypt ($P = 3.8 \times 10^{-7}$), the colonic crypt ($P = 5.3 \times 10^{-3}$) and the caecal crypt ($P = 0.003$, Wilcoxon rank sum exact tests).

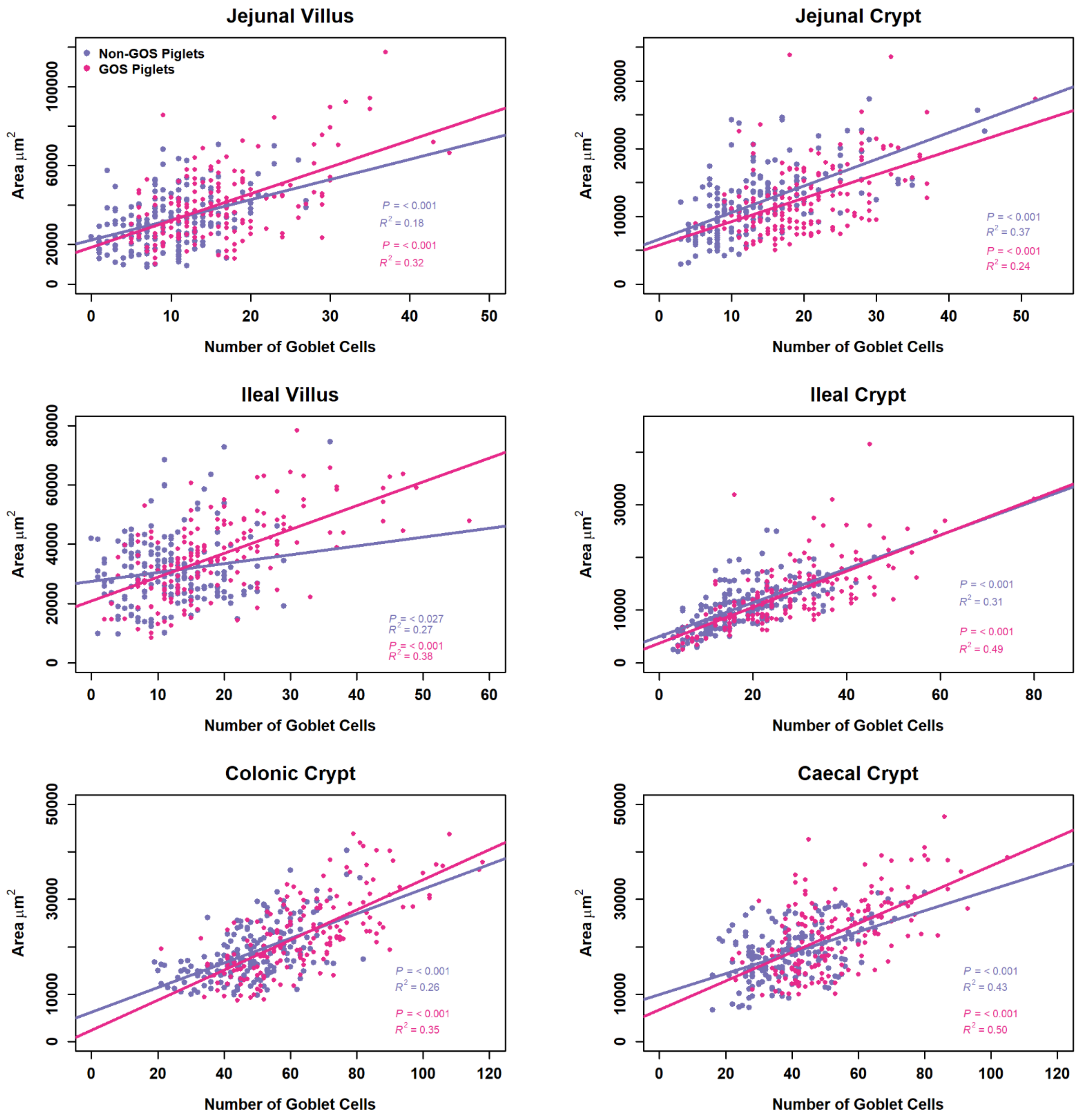

Supplement: Supplementary file 1 [file animals-13-00230-s001.zip › animals-2095088-supplementary.pdf]
